# Coexistence of glutamatergic spine synapses and shaft synapses in substantia nigra dopamine neurons

**DOI:** 10.1038/srep14773

**Published:** 2015-10-05

**Authors:** Miae Jang, Ki Bum Um, Jinyoung Jang, Hyun Jin Kim, Hana Cho, Sungkwon Chung, Myoung Kyu Park

**Affiliations:** 1 Department of Physiology, Sungkyunkwan University School of Medicine 300 Chunchun-dong, Jangan-ku, Suwon, 440-746, Korea; 2 Center For Molecular Medicine, Samsung Biomedical Research Institute, 300 Chunchun-dong, Jangan-ku, Suwon, 440-746, Korea

## Abstract

Dopamine neurons of the substantia nigra have long been believed to have multiple aspiny dendrites which receive many glutamatergic synaptic inputs from several regions of the brain. But, here, using high-resolution two-photon confocal microscopy in the mouse brain slices, we found a substantial number of common dendritic spines in the nigral dopamine neurons including thin, mushroom, and stubby types of spines. However, the number of dendritic spines of the dopamine neurons was approximately five times lower than that of CA1 pyramidal neurons. Immunostaining and morphological analysis revealed that glutamatergic shaft synapses were present two times more than spine synapses. Using local two-photon glutamate uncaging techniques, we confirmed that shaft synapses and spine synapses had both AMPA and NMDA receptors, but the AMPA/NMDA current ratios differed. The evoked postsynaptic potentials of spine synapses showed lower amplitudes but longer half-widths than those of shaft synapses. Therefore, we provide the first evidence that the midbrain dopamine neurons have two morphologically and functionally distinct types of glutamatergic synapses, spine synapses and shaft synapses, on the same dendrite. This peculiar organization could be a new basis for unraveling many physiological and pathological functions of the midbrain dopamine neurons.

Glutamatergic inputs to the midbrain dopamine neurons carry reward-related information and thereby play a key role in many brain functions including action selection, reinforcement learning, voluntary motor control, and drug addiction^1^,^2^,^3^,^4^. Several brain regions provide glutamatergic afferent inputs into the midbrain dopamine neurons, including the subthalamic nucleus, pedunculopontine nucleus, laterodorsal tegmentum, and prefrontal cortex^5^,^6^,^7^. At the cellular level, as major excitatory inputs, glutamatergic fibers in the autonomously firing dopamine neurons can trigger a variety of cellular events including Ca^2+^ signals that are important for synaptic actions and plasticity, and ultimately regulate tonic firing and produce proper types of phasic discharges^8^. The tonic firing rate determines ambient dopamine levels of the brain^9^,^10^,^11^, whereas the phasic firing known as bursts seems to evoke dopamine surges and encode reward prediction error, which is a critical mediator of reinforcement learning^11^,^12^,^13^. Although the midbrain dopamine neurons clearly receive many glutamatergic inputs from several distinct regions of the brain, it is not clear how information from these distinct afferent fibers is integrated and translated into dopamine neurons. This poor understanding is, partly, attributed to the lack of detailed information about the morphology, distribution, and biochemical/electrical properties of single glutamatergic synapses in the dopamine neurons.

In general, large central neurons such as hippocampal pyramidal neurons, cerebellar Purkinje neurons, and many cortical pyramidal neurons, form glutamatergic synapses predominantly on small membranous protrusions called dendritic spines for compartmentalized signal processing^14^,^15^. However, there is currently no clear evidence for functioning dendritic spines in the midbrain dopamine neurons. Although a few papers provided a morphological evidence for the presence of dendritic spines in some types of ventral tegmental dopamine neurons^16^,^17^, they did not provide detailed and functional characteristics of single dendritic spines. Therefore, to date, most experiments have been performed on the bases that dendrites of dopamine neurons are largely aspiny or have few spines^18^,^19^,^20^,^21^,^22^,^23^. Given the importance of glutamatergic afferents into the midbrain dopamine neurons, there is an urgent need to establish the morphological and functional bases of glutamatergic synapses in the midbrain dopamine neurons at the single synapse level.

Therefore, in the present study, we used high-resolution two-photon confocal microscopy in the mouse midbrain slices, to examine morphological features of dendrites and glutamatergic synapses in the nigral dopamine neurons. We provide the first evidence that the midbrain dopamine neuron is a particular type of neuron that possesses a substantial number of two morphologically and functionally distinct glutamatergic synapses, spine synapses and shaft synapses, on the same dendrite. This characteristic organization of glutamatergic synapses could be an important base for further studies of dopamine neuron functions.

## Results

### Morphological features of dendrites in the nigral dopamine neurons

**Figure 1:**
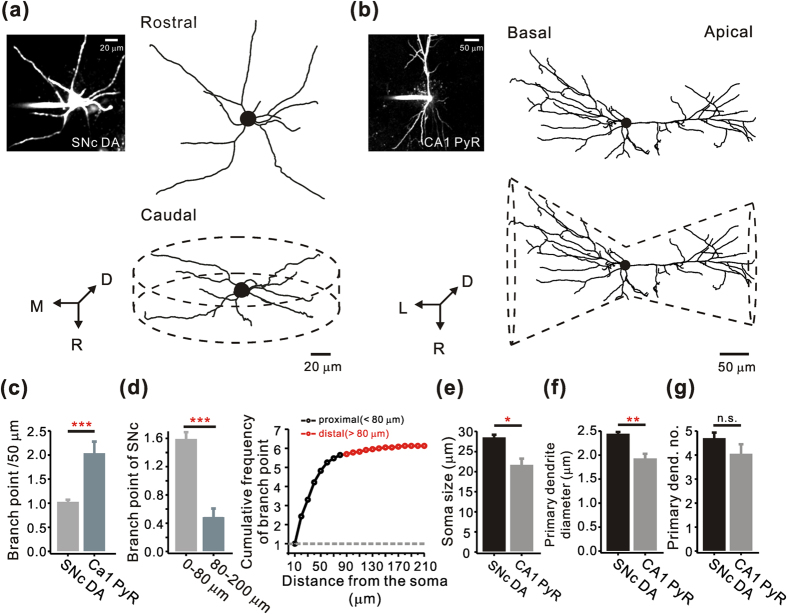
Comparison of dendritic arborization in the SNc dopamine neurons and hippocampal CA1 pyramidal neurons. (**a**,**b**) Z-projection of a nigral dopamine neuron and a CA1 pyramidal neuron, labeled with cascade blue. Left, fluorescence image; Right, reconstructed projection image; Bottom, 3D-reconstructed image. (**c**) Average numbers of branch points in SNc dopamine neurons and CA1 pyramidal neurons (SNc, from 80 dendrites in 34 cells; CA1, from 23 dendrites in 5 cells). (**d**) Left, distribution of branch points in the proximal and distal dendrites in SNc dopamine neurons (proximal, <80 μm from the soma, n = 80; distal, >80 μm from the soma, n = 35). Right, cumulative frequency plot of branch points along the dendrite of SNc dopamine neurons (n = 115). The cumulative frequency is normalized by branch point number from 0 (soma) to 10 μm. (**e**) Comparison of soma size between SNc dopamine neurons and CA1 pyramidal neurons. (SNc, n = 34; CA1, n = 5). (**f**,**g**) Comparison of diameters and numbers of primary dendrite between SNc dopamine neurons and CA1 pyramidal neurons. Primary dend. no. indicates primary dendrite number. (SNc, primary dendrite diameter, n = 140; primary dendrite number, n = 34; CA1, primary dendrite diameter, n = 18; primary dendrite number, n = 5). *p < 0.05; **p < 0.01; ***p < 0.001, Unpaired *t*-tests. *, **, and *** indicate statistical significance. Error bars represent SEM.

The majority of isolated dopamine neurons from the midbrain can be characterized by a large soma and multiple long dendrites with a simple dendritic arborization^24^,^25^,^26^,^27^. To better understand the number, orientation, and arborization pattern of the dendrites of dopamine neurons in intact midbrain tissue, we used midbrain slices from tyrosine hydroxylase(TH)-eGFP transgenic mice in which dopamine neurons can be identified by expression of enhanced green fluorescent protein driven by the TH promoter. Although it was recently reported that dopamine-specific transgenic mouse line exhibits dramatic non-dopamine specific expression patterns in some parts of the VTA nuclei^28^, in our TH-eGFP mouse line all of GFP-expressing neurons (n = 12) recorded from the substantia nigra pars compacta (SNc), showed the typical electrophysiological properties of dopamine neurons (Fig. S1). Two-photon laser scanning confocal microscopy was employed to image dendritic trees spreading several hundred micrometers deep from the midbrain slice surface. As shown in Fig. 1a, the whole dendrites of a dopamine neuron in the SNc filled large cylindrical spatial domains approximately 200 μm × 50 μm in diameter and 300 μm thick, randomly ramifying in all directions from the central soma. Such dendritic arbors may receive widely distributed synaptic inputs, similar to reticular neurons and pallidal neurons^29^. To compare the dendritic arborization of the dopamine neurons with another central neuron, we also imaged hippocampal CA1 pyramidal neurons from the same mice. The CA1 pyramidal neurons extended their dendrites in two different directions and thereby formed two typical dendritic arbors: the apical and basal dendritic compartments (Fig. 1b). This corresponds to a bidirectional radiation, and may be the best configuration for complex information processing driven by two geometrically different afferent inputs^30^. To compare the complexity of dendritic arborization quantitatively, we counted the number of branching points in the dendrites^31^,^32^. The number of branch-points of the nigral dopamine neurons was less than half that of the CA1 pyramidal neurons (SNc = 0.98 ± 0.06; CA1 = 1.98 ± 0.26; *p* = 0.001; Fig. 1c), indicating a simpler morphological feature of the nigral dopamine neurons despite the wider distribution of the dendritic trees (Fig. 1a,b). Moreover, when we compared the numbers of branch-points between the proximal (<80 μm from the soma) and distal (>80 μm from the soma) dendritic regions, the branch-points were mainly found within the proximal dendritic regions in the dopamine neurons (proximal dendritic region = 1.56 ± 0.11; distal dendritic region = 0.45 ± 0.13; *p* = 0.001; Fig. 1d), suggesting that the dopamine neurons might integrate information in a concentric manner throughout the whole dendritic tree. In contrast, the synaptic information in the CA1 pyramidal neurons seems to be integrated in a compartmentalized way, first in the highly branching distal dendrites and then in the proximal dendrites^33^.

In many neurons, the diameter of the primary dendrites tends to be proportional to the diameter of the soma^34^,^35^. Thus, the nigral dopamine neurons with a larger soma size showed a thicker primary dendrite diameter compared with the CA1 pyramidal neurons (soma size, SNc = 28.21 ± 0.98; CA1 = 21.39 ± 1.83; *p* = 0.016; primary dendrite diameter, SNc = 2.42 ± 0.06; CA1 = 1.90 ± 0.12; *p* = 0.004; Fig. 1e,f). However, there was no significant difference in the number of primary dendrites extending from the soma between the nigral dopamine neurons and CA1 pyramidal neurons (SNc = 4.64 ± 0.28; CA1 = 4 ± 0.44; *p* = 0.406; Fig. 1g), as previously described in acutely dissociated dopamine neurons^27^.

### Morphological identification of dendritic spines in the nigral dopamine neurons

**Figure 2:**
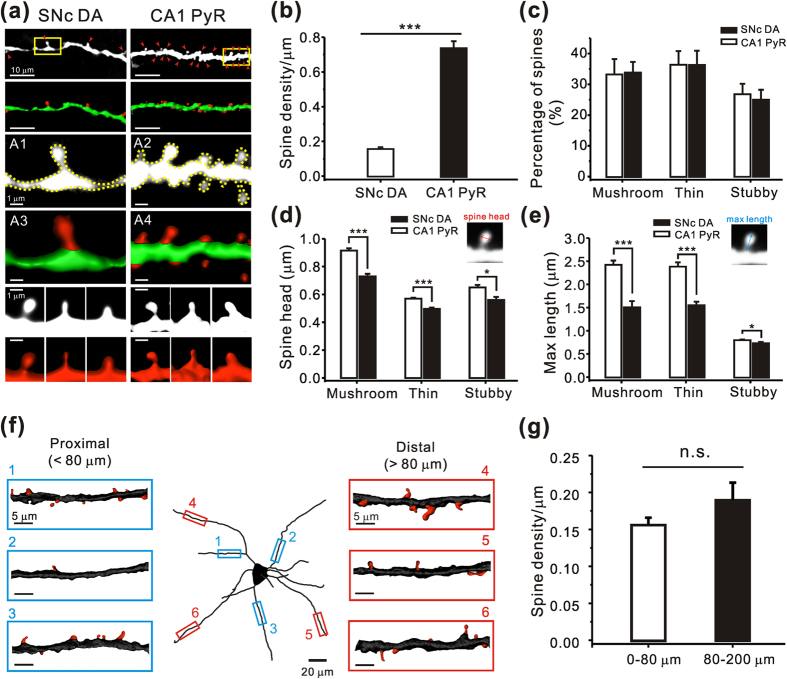
Morphological analysis of dendritic spines in the SNc dopamine neurons. (**a**) Monochrome and three-dimensional (3D) reconstructed images of the dendritic segment (green and red images; shafts are green and spines are red). Red arrowheads indicate dendritic spines. A1-4, monochrome and 3D-reconstructed images of the yellow boxed regions in the SNc dopamine neurons and CA1 pyramidal neurons, respectively, at high magnification. Lower panels show monochrome and 3D-reconstructed images of mushroom, thin, and stubby-type spines in SNc dopamine neurons and CA1 pyramidal neurons. (**b**) Mean spine densities of SNc dopamine neurons and CA1 pyramidal neurons (SNc, from 80 dendrites in 34 cells; CA1, from 23 dendrites in 5 cells). (**c**) Distribution of each spine subtype in SNc dopamine neurons and CA1 pyramidal neurons (SNc, mushroom = 189; thin = 174; stubby = 125; CA1, mushroom = 42; thin = 44; stubby = 31). (**d**,**e**) Comparison of mean spine head diameters and maximum longitudinal lengths in three typical subtypes of spines in SNc dopamine neurons (mushroom = 189; thin = 174; stubby = 125) and CA1 pyramidal neurons (mushroom = 42; thin = 44; stubby = 31). (**f**) Distribution of dendritic spines in SNc dopamine neurons. Each segment of dendritic regions (marked as blue [proximal] and red [distal] rectangles in the reconstructed projection image of a SNc neuron) is expanded in the blue and red boxed insets, in which dendritic shafts are seen with dendritic spines in red. (**g**) Average spine densities in the proximal (0–80 μm; n = 80) and distal (80–200 μm; n = 35) dendritic regions. * and *** represent *p* < 0.05 and *p* < 0.001, respectively, by unpaired *t*-test. Error bars represent SEM.

Next, to explore whether there are spine-like structures in the dendrites of the dopamine neurons, we performed detailed two-photon confocal imaging in the dendritic regions in live midbrain slices. Intracellular injection of Alexa fluor 594 or Cascade blue with a whole-cell patch-clamp microelectrode into the dopamine neurons, mostly in the SNc region revealed their dendritic structures more clearly. As shown in Fig. 2a, we surprisingly found a substantial number of spine-like structures throughout the whole dendritic tree in the dopamine neurons. Imaging of the dendritic trees of hippocampal CA1 pyramidal neurons in the same mice showed that the dendritic spines were more-densely spaced, and their general appearance was much similar to those found in the nigral dopamine neurons (Fig. 2a). For statistical analysis, the densities and lengths of individual spines were measured from 34 nigral dopamine neurons in 26 mice and 5 hippocampal CA1 pyramidal neurons in 4 mice (Fig. 2a–e). Although the number of spine-like structures in nigral dopamine neurons was much lower than that in CA1 pyramidal neurons (SNc, 0.15 ± 0.009; CA1, 0.73 ± 0.04; *p* = 0.001; Fig. 2b), the general shapes of the dendritic spines (as shown in the fluorescence images and 3D rendering images in the bottom of Fig. 2a) and the distributions of individual spine types found in the dendrites of the nigral dopamine neurons (Fig. 2c) were similar to those of CA1 pyramidal neurons (Fig. 2a–c). The three common types of dendritic spines—mushroom, thin, and stubby— were found in both neurons with a similar proportion (SNc, mushroom = 33.21 ± 4.99; thin = 36.38 ± 4.41; stubby = 26.78 ± 3.38; CA1, mushroom = 33.85 ± 3.43; thin = 36.31 ± 4.62; stubby = 24.99 ± 3.23; Fig. 2c). However, the exact dimensions of the spines were significantly different for all three types of spines, not only in head diameters (mushroom, SNc = 0.91 ± 0.01; CA1 = 0.73 ± 0.01; *p* = 0.001; thin, SNc = 0.57 ± 0.006; CA1 = 0.49 ± 0.009; *p* = 0.001; stubby, SNc = 0.65 ± 0.01; CA1 = 0.56 ± 0.02; *p* = 0.013; Fig. 2d), but also in the maximum longitudinal lengths (mushroom, SNc = 2.42 ± 0.09; CA1 = 1.50 ± 0.07; *p* = 0.001; thin, SNc = 2.38 ± 0.09; CA1 = 1.55 ± 0.07; *p* = 0.001; stubby, SNc = 0.79 ± 0.01; CA1 = 0.73 ± 0.02; *p* = 0.044; Fig. 2e), despite the similar shape of each type of dendritic spine (Fig. 2a). In general, the head diameters and maximum longitudinal lengths of dendritic spines in SNc dopamine neurons were larger than those of CA1 pyramidal neurons (Fig. 2d,e). In addition, although spine-like structures in nigral dopamine neurons were found in all regions of the dendritic tree, the spines were not uniformly distributed along a dendrite (Fig. 2f) and the spine density measured in each dendritic segment (< 200 μm) ranged from 0.04/μm to 0.42/μm. However, when we compared the densities of spine-like structures between the proximal and distal dendritic regions, there was no significant difference (proximal dendrites = 0.15 ± 0.01; distal dendrites = 0.18 ± 0.09; *p* = 0.188 Fig. 2g). To rule out the possibility that transgenes under control of TH may affect dendritic spine genesis, SNc dopamine neurons in wild-type mice were analysed with TH-immunostaining after morphological and electrical experiments (Fig. S2). Dendritic spines were also found in wild-type mice with a similar density in TH-eGFP mice (Fig. S2). Therefore, our data indicate that SNc dopamine neurons possess dendritic spines and appear to be categorized as low-density spiny neurons according to the previously described classification^36^.

### Molecular identification of glutamatergic synapses in the dendrite of the dopamine neurons

**Figure 3:**
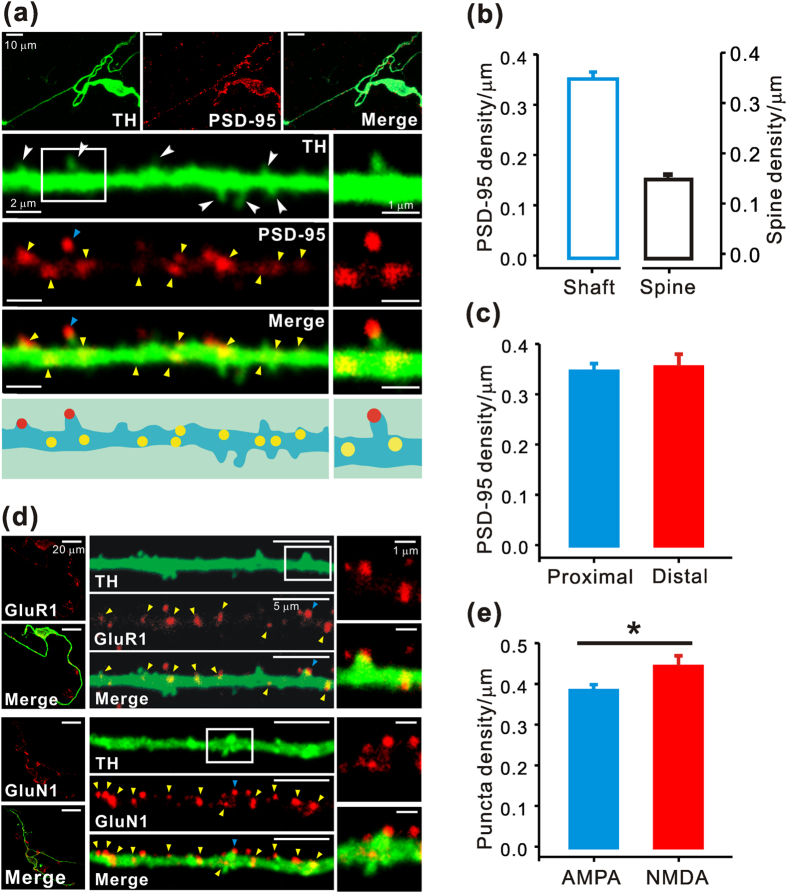
Molecular identification of glutamatergic synapses in dendrites of the SNc dopamine neurons. (**a**) Double immunostaining with antibodies specific for TH and PSD-95 in very gently dissociated dopamine neurons. Top, TH-positive (green), PSD-95-labeled (red), and overlapped images of a dopamine neuron (merge). Second to fourth lanes, expanded fluorescence images from a representative dendritic region. Bottom, images showing a dendritic shaft and PSD-95 synaptic sites. Arrowheads indicate PSD-95 fluorescence puncta in spines (blue) and shafts (yellow). Right, high-magnification images of the dendritic segment marked by a white box (left) show PSD-95 puncta in both dendritic spines and shafts. (**b**) PSD-95 density in the dendritic shaft, calculated from 12 dendrites in 10 cells. Error bar indicates SEM. Right, the spine density was redrawn from Fig. 2b,c for comparison. **(c)** Densities of PSD-95 puncta in the proximal dendritic shafts (<80 μm from the soma; n = 12) and distal dendritic shafts (>80 μm from the soma, n = 12). Error bars indicate SEM. (**d**) Distribution of AMPA receptors and NMDA receptors in the dendritic spines and shafts of SNc dopamine neurons. Double immunostaining of dissociated dopamine neurons with TH and either GluR1 for AMPAR or GluN1 for NMDAR. TH (green), GluR1 or GluN1 (red), and their overlapped images (merge) are presented. Arrowheads indicate GluR1 and GluN1 fluorescence puncta in spines (blue) and dendritic shafts (yellow). Dendritic regions containing a single spine marked by the white boxes are presented on the right. **(e)** GluR1 puncta (from 27 dendrites of 18 cells) and GluN1 puncta (from 17 dendrites of 10 cells) measured only in the dendritic shaft. *p < 0.05 by unpaired *t*-tests. Error bars indicate SEM.

Excitatory glutamatergic synapses can be characterized by a morphological and functional specialization of the postsynaptic membrane called postsynaptic density (PSD), which is usually located at the tip of the dendritic spines in most large central neurons or at the shaft synapses on the dendrites of aspiny neurons^37^,^38^. The PSD contains glutamate receptors such as AMPA receptors (AMPAR) and NMDA receptors (NMDAR)^39^ and many associated signaling and structural molecules including PSD-95 and calcium calmodulin-dependent kinase II^40^,^41^. We examined the localization of PSD-95, which is used as a marker for glutamatergic synapses, in the dendrites of the dopamine neurons. During these experiments we found that very gently dissociated dopamine neurons from SNc slice fragments showed much clearer staining results than the dopamine neurons embodied in the midbrain slices themselves, and PSD-95 fluorescence was clearly seen as puncta along a dendritic tree. PSD-95 puncta were present not only on the dendritic spines, but also on the dendritic shafts of the dopamine neurons (Fig. 3a). The dopamine neuron was identified by positive staining with TH antibody, and spine-like structures were also seen in the fluorescence images (white arrows in Fig. 3a). The part of the dendritic region containing a spine (the white box in Fig. 3a) is expanded on the right and PSD-95 puncta are clearly present both on the spine and on dendritic shafts. These data strongly suggest that the dopamine neurons possess two morphologically distinct glutamatergic synapses on the same dendritic region, a spine synapse and a shaft synapse. The number of PSD-95 puncta counted only in the dendritic shafts (PSD-95 density = 0.35 ± 0.01; Fig. 3b) was approximately 2 times higher than the number of the spine-like structures in the dopamine neurons (Fig. 2b), indicating that there are more than twice as many shaft synapses than spine synapses in the dopamine neurons. There was no significant difference between the densities of PSD-95 puncta in the proximal and distal dendritic regions (proximal dendrites = 0.34 ± 0.01; distal dendrites = 0.35 ± 0.02; *p* = 0.747; Fig. 3c). Therefore, the PSD-95 puncta seem to be evenly distributed throughout the whole dendritic tree and the glutamatergic shaft synapses appear to exist approximately every 2.88 ± 0.09 μm.

If the PSD-95 puncta observed on the dendritic spines and shafts are real glutamatergic synapses, glutamate receptors must be expressed on the same membrane areas. Therefore, we examined the expression of glutamate receptors on the spine-like structures and dendritic shafts by immunostaining with glutamate receptor antibodies: GluR1 for AMPAR, and GluN1 for NMDAR^42^,^43^. Interestingly, as shown in Fig. 3d, fluorescence puncta of both GluR1 and GluN1 were found in the dendritic spines as well as the dendritic shafts. Typical TH-positive dopamine neurons are seen in the left column of Fig. 3d, together with fluorescence images showing puncta of glutamate receptors. The right panel shows one part of dendritic region and more magnified images containing a single spine (from the white box), in which GluR1 and GluN1 are clearly seen as puncta on the dendritic shafts as well as the dendritic spines. The density of GluR1 and GluN1 puncta in the dendritic shafts (Fig. 3e) was similar to that of PSD-95 (GluR1 = 0.38 ± 0.01; *p* = 0.220; GluN1 = 0.44 ± 0.02; *p* = 0.006; Fig. 3e), although there was a slight difference between the densities of AMPAR and NMDAR puncta (GluR1 = 0.38 ± 0.01; GluN1 = 0.44 ± 0.02; *p* = 0.029; Fig. 3e). The same results were obtained with surface-specific antibodies for GluR1 and GluN1 in non-permeabilized dopamine neurons (Fig. S3). All these data strongly suggest that spine synapses and shaft synapses in dopamine neurons contain both AMPA receptors and NMDA receptors together.

### Functional identification of the glutamatergic spine synapses in the nigral dopamine neurons

**Figure 4:**
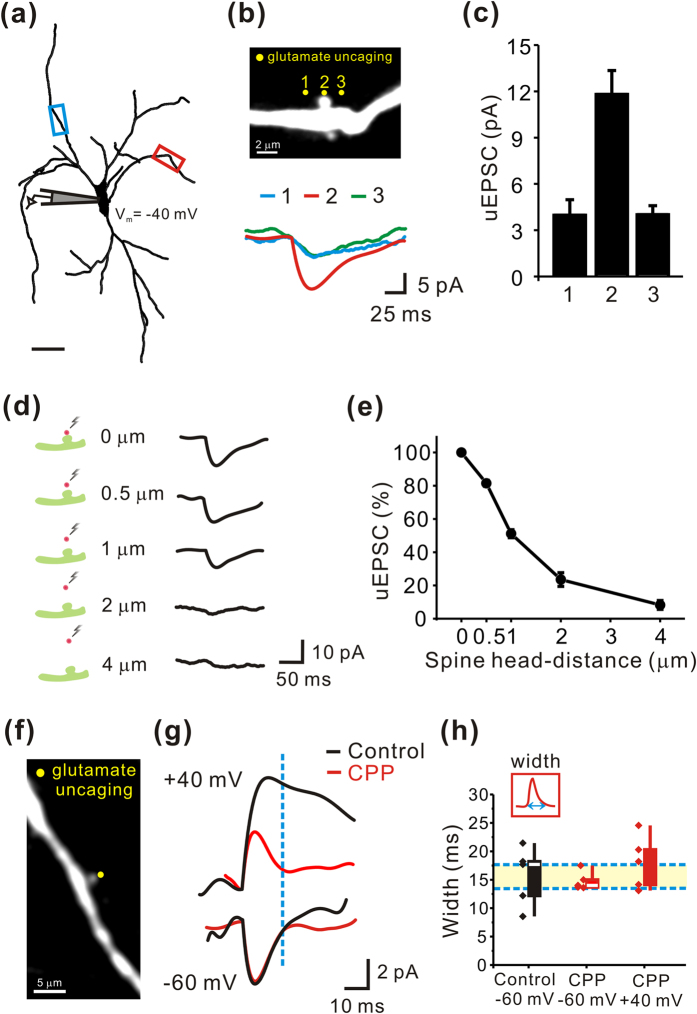
Functional identification of glutamatergic spine synapses in the SNc dopamine neurons. Single uEPSCs from a single dendritic spine were measured using two-photon glutamate uncaging. (**a**) Reconstructed image of a recorded neuron filled with Alexa Fluor 594 with two recording sites. (**b**) Top, high-magnification image of the target dendritic segment indicated by the red box in a. Yellow dots indicate uncaging sites. Bottom, representative traces of uncaging-evoked EPSCs. (**c**) Summary of recordings from 4 spines in 4 cells. (**d**) Left, illustration of the target dendrite from the red box indicated in a. Red dots indicate the uncaging locations with distance from the dendritic spine head. Right, representative traces of uncaging-evoked EPSCs. uEPSCs decreased as the uncaging spot was moved away from the spine head. (**e**) The first point corresponds to data obtained by uncaging on the spine head. Each subsequent point was normalized to this point (n = 5). (**f**) Monochrome image magnifying the target dendritic segment indicated by the blue box in a. (**g**) Representative traces of uncaging-evoked EPSCs in the absence (black) and presence (red) of CPP. (**h**) Summary of the widths of uEPSCs. AMPAR EPSCs were recorded at −60 mV in the absence (black, n = 5) or presence (red, n = 5) of CPP and at +40 mV in the presence of CPP (red, n = 5). The amplitudes of the NMDAR EPSCs were measured at 14 ms after the onset (blue dotted line), when the AMPAR EPSCs had returned to baseline.

**Figure 5:**
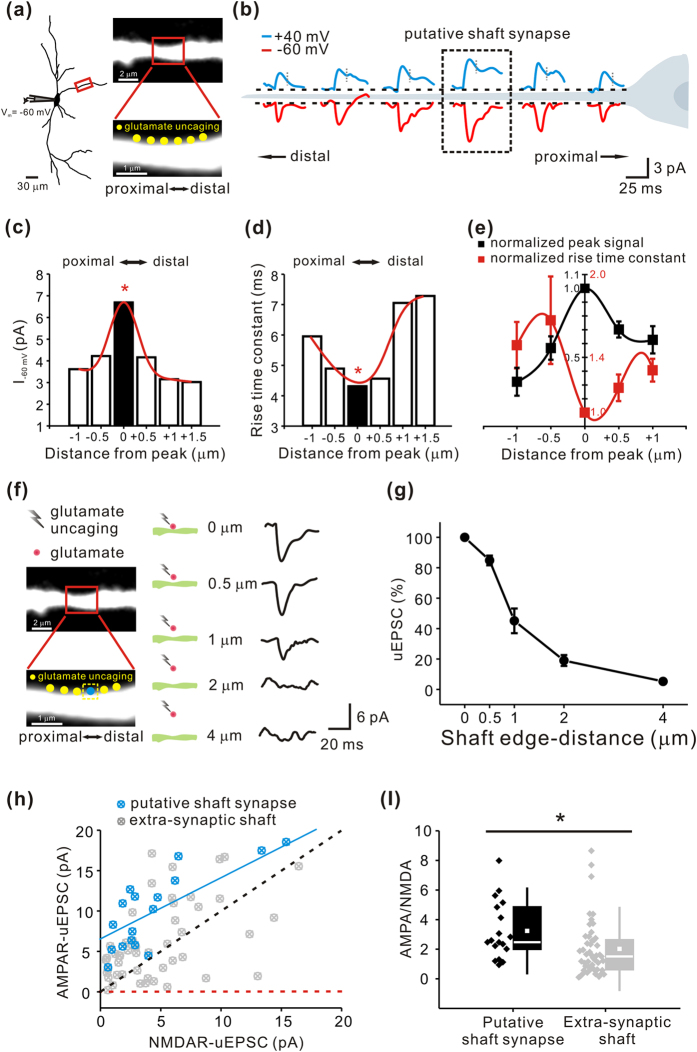
Functional identification of glutamatergic shaft synapses in the SNc dopamine neurons. AMPAR and NMDAR uEPSCs from a single shaft synapse were measured using two-photon glutamate uncaging. (**a**) Left, reconstructed image of a recorded dopamine neuron filled with Alexa Fluor 594. Top, monochrome image of the target dendritic region magnified from the red boxed region. Bottom, local glutamate uncaging was serially performed along an aspiny region of a dendrite (yellow dots). (**b**) A series of AMPAR and NMDAR uEPSCs are presented schematically. To identify a functional shaft synapse, local glutamate uncaging was serially performed along a dendrite. AMPAR and NMDAR uEPSCs were evoked at holding potentials of −60 mV (red) and +40 mV (blue), respectively, using 1 ms uncaging pulses. The dotted box in the middle indicates a putative shaft synapse. The gray dashed lines indicate measuring points for NMDAR uEPSC amplitudes. (**c**,**d**) Peak amplitudes and rise time constants of AMPAR uEPSCs measured from the representative traces in (**b**). Red *indicates a putative shaft synapse. (**e**) Normalized peak amplitudes (AMPAR uEPSC) and rise time constants (AMPAR uEPSC) are statistically plotted as a function of dendritic distance around a central putative shaft synapse (n = 7). (**f**) Left, the blue dot covered by a yellow dotted box indicates a putative shaft synapse from (**b**). Middle, illustrations of the target dendritic region with uncaging sites marked as red dots. Right, representative traces of uncaging-evoked EPSCs. (**g**) The first point corresponds to uncaging on the edge of a putative shaft synapse. The uEPSCs decrease rapidly with distance from the dendritic shaft surface (n = 5). (**h**,**I**) Scatter plot for AMPAR and NMDAR uEPSCs and scatter and related box plots for the ratios of AMPAR/NMDAR currents in putative shaft synapses and extra-synaptic sites (putative shaft synapses, R = 0.82094, n = 17; extra-synaptic shaft, R = 0.501161, n = 48). The black dashed line is the regression line for a linear relationship between AMPAR and NMDAR uEPSC amplitudes. The red dashed line marks the expected position in the absence of AMPAR uEPSCs. The blue line is the fitted AMPAR/NMDAR uEPSC ratio. *p < 0.05. Error bars indicate SEM.

**Figure 6:**
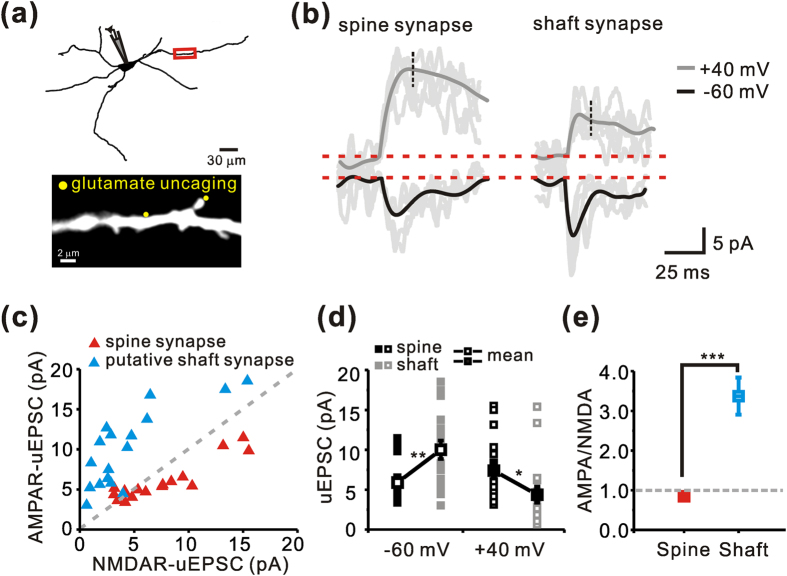
Difference in AMPAR/NMDAR current ratios between spine synapses and shaft synapses in the SNc dopamine neurons. (**a**) Top, reconstructed image of a recorded dopamine neuron filled with Alexa Fluor 594. Bottom, monochrome image of the target dendritic region expanded from the red boxed region. The yellow dots indicate the locations of two-photon glutamate uncaging for a shaft synapse and a spine synapse. (**b**) Representative traces of AMPAR and NMDAR uEPSCs at holding potentials of −60 mV (black) and +40 mV (dark gray) in the spine and putative shaft synapse, respectively. Five individual trials and the corresponding average traces (black and dark gray traces) are shown at a spine and shaft synapse. NMDAR uEPSC amplitudes were taken 14 ms after the EPSC onset (black dashed lines). (**c**) Scatter plot showing the relationship between AMPAR uEPSCs and NMDAR uEPSCs in spine (red triangles) and shaft (blue triangles) synapses. The gray dashed line is the regression line for a linear relationship. (**d**) Amplitudes of uEPSCs from AMPAR (at −60 mV) in spines were smaller in shaft synapses, whereas the amplitudes of uEPSCs from NMDAR (at +40 mV) were larger (spine synapse, n = 17; shaft synapse, n = 17). (**e**) Difference in AMPA/NMDA current ratios between spine and shaft synapses (spine, n = 17; shaft, n = 17). *p < 0.05; **p < 0.01; ***p < 0.001 by unpaired *t*-tests. Error bars indicate SEM.

**Figure 7:**
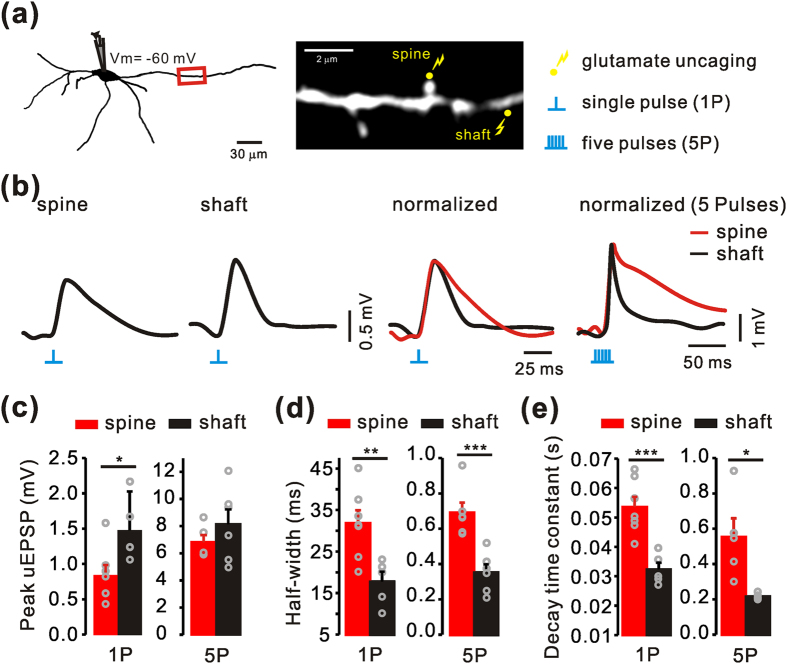
Comparison of single and multiple synaptic potentials between spine synapses and shaft synapses in the SNc dopamine neurons . (**a**) Left, reconstructed image of a recorded dopamine neuron filled with Alexa Fluor 594. The red box indicates the target dendritic region. Right, monochrome image of the target dendritic region expanded from the red boxed region. Yellow dots indicate the sites of uncaging for a spine synapse and a shaft synapse. A single spine synapse and shaft synapse were stimulated by one single (1P) or repetitive glutamate uncaging with 5 pulses over 1 ms at 10-ms intervals (5P), respectively. (**b**) Left, representative traces of uEPSPs from a single spine synapse and shaft synapse by single glutamate uncaging pulses. Right, normalized traces of each uEPSP for 1P or 5P between the spine and shaft synapses. (**c**–**e**) Comparison of the amplitudes of the uEPSP, half-widths, and the durations of decay time constant (τ) between spine (1P, n = 7; 5P, n = 5) and shaft synapses (1P, n = 5; 5P, n = 5). *, ** and *** represent *p* < 0.05, *p* < 0.01, and *p* < 0.001 by unpaired t-test, respectively. Data are represented as mean ± SEM.

Next we asked whether the dendritic spines present on the dendrites of the dopamine neurons can function as a real glutamatergic synapse. We addressed this question using whole-cell patch-clamp recordings combined with two-photon glutamate uncaging techniques in live midbrain slices. Dopamine neurons were filled with the red-fluorescence dye Alexa Fluor 594 (30 μM) through the patch pipette to visualize the dendritic shafts and spines. To detect very small current changes from a single synapse, the proximal dendritic regions within 100 μm from the soma were intensively investigated (Figs 4, 5, 6, 7).

To stimulate only a single synapse, we used a brief two-photon laser photolysis (1 ms with 720 nm) in the presence of 2.5 mM MNI-caged-L-glutamate^44^. This caged glutamate compound evoked excitatory postsynaptic currents (uEPSCs) in most cases (Fig. 4b,f). To verify whether the uEPSC was generated from only a single spine synapse under our photolysis condition, we moved an uncaging spot around the target spine in a horizontal (Fig. 4b,c) and vertical direction (Fig. 4d,e). Glutamate uncaging on the left or right side of a target spine head evoked much smaller currents in contrast to the currents recorded upon direct stimulation of the spine head itself (Fig. 4b,c). Similarly, a two-photon glutamate uncaging spot was vertically moved away from the tip of the spine head (Fig. 4d) and the uEPSCs decreased with increasing distance from the spine head (Fig. 4e). The maximal responses could be obtained from the very tip of the spine head up to a distance of 0.5 μm. Thus, in our experimental conditions, uncaging sites were maintained within a limited range of less than 0.5 μm from the spine head, which corresponds to the standard uncaging locations shown in other laboratories^45^,^46^. Since glutamatergic synapses in the dopamine neurons seem to express both NMDAR and AMPAR (Fig. 3), we tried to separately record AMPAR and NMDAR currents from a single spine synapse using this technique. For this, we evoked uEPSCs in the same spine from two different holding potentials at −60 mV and +40 mV, respectively. Because of the different activation and desensitization kinetics of NMDAR and AMPAR^47^,^48^, we were able to separately measure AMPAR and NMDAR uEPSCs (Fig. 4f,g). At −60 mV AMPAR currents can be purely evoked, but at +40 mV both AMPAR and NMADR currents are activated. Application of 10 μM CPP, a NMDAR antagonist (red traces in Fig. 4g,h) affected uEPSCs evoked at +40 mV, but not those evoked at −60 mV (Fig. 4g). AMPAR uEPSCs returned to basal levels within 14 ms not only at −60 mV (14.76 ± 0.72; Fig. 4h) but also +40 mV as shown in the red trace in Fig. 4g. Therefore, without blockade of AMPAR blockers, we could estimate NMDA currents at +40 mV by measuring currents at 14 ms after the onset of the currents (blue dotted line in Fig. 4g). Most NMDA uEPSCs were obtained under this condition in the absence of AMPA receptor blockers and measuring points were marked as a dotted line. From these experiments, we could conclude that the dendritic spines of nigral dopamine neurons contain functioning AMPAR and NMDAR together.

### Functional identification of the glutamatergic shaft synapses in the nigral dopamine neurons

It was now clear that dendritic spine-like structures could function as a glutamatergic synapse in dopamine neurons, but it was not clear whether the PSD-95 puncta found on the dendritic shaft without any membranous protrusion represented a functioning glutamatergic synapse. Although there were similar distributions of PSD-95, GluR1, and GluN1 puncta along the smooth surface of the whole dendrite (Fig. 3), real glutamatergic synapses should not only have glutamate receptors, but should also respond to glutamate. Since the local glutamate uncaging on the aspiny regions of the dendritic shaft might activate not only glutamate receptors on postsynaptic membranes but also glutamate receptors present on extrasynaptic sites, it may not be easy to identify the site of functioning shaft synapses with a simple local glutamate uncaging. Therefore, to identify active single synapses at the aspiny region of a dendrite, we applied five or six consecutive but tightly spaced pulses along a smooth dendritic segment over several micrometers (yellow dots in Fig. 5a). As estimated from the PSD-95 and glutamate receptor puncta densities shown in Fig. 3, there could be one shaft synapse every 2.8 μm along a dendrite. If we perform local glutamate uncaging, there is a chance of finding one shaft synapse within this range. Therefore, we might expect to find a hot spot (a synaptic site on dendritic shafts) around which the evoked current decreases in a bell shape. During these experiments, to selectively measure AMPAR and NMDAR uEPSCs, we maintained holding potentials at different levels of either −60 mV or +40 mV, as previously described (Fig. 4g). AMPAR-mediated uEPSCs were recorded at −60 mV and NMDAR-mediated uEPSCs were recorded at +40 mV in the voltage-clamp mode. The data shown in Fig. 5b represent a case in which AMPAR and NMDAR EPSCs synchronously decreased around the hot spot in the expected bell shape. AMPAR and NMDAR uEPSCs also peaked at the same site. This indicates that the hot spot represented as a dotted rectangle in Fig. 5b could be the site of a glutamatergic shaft synapse. The amplitudes of AMPAR and NMDAR uEPSCs changed similarly around the hot spot (Fig. 5b). When we analyzed AMPA uEPSCs in the hot spots of 7 different neurons, in all cases the amplitudes were highest and the rising times were also fastest (Fig. 5c–e). After determining the site of a shaft synapse, the two-photon glutamate uncaging site was moved vertically from the edge of the synapse surface (Fig. 5f). The uEPSCs decreased with distance from the membrane and the maximal responses were obtained within ~0.5 μm (Fig. 5f,g), very similar to the spine synapses (Fig. 4d).

Since AMPAR and NMDAR uEPSCs were measured at the same site as a pair, these two currents from putative synaptic sites and extrasynaptic sites can be compared by 2D-distribution plots (Fig. 5h). Examination of the distributions of 48 individual uEPSCs from extrasynaptic sites (gray crossed open dots) and 17 uEPSCs from putative synaptic sites (blue crossed open dots) revealed a big difference between the distribution patterns: the dots from extrasynaptic sites were scattered widely and randomly, whereas the dots from putative synaptic sites were arranged linearly with a higher proportion of AMPAR currents. The AMPAR/NMDAR uEPSC ratio (fitted blue line) seems to be similar for putative shaft synapses despite the various amplitudes of the uEPSCs. Although the AMPAR currents were several fold larger than the NMDAR currents in this putative shaft synapses, the currents recorded at extrasynpatic regions seemed to vary. When we compared the average values of AMPAR/NMDAR current ratios, the ratio of AMPAR/NMDAR uEPSCs in the shaft synapses was significantly greater than that recorded at extra-synaptic sites (shaft synapse = 3.36 ± 0.47; extra-synaptic shaft = 2.02 ± 0.27; *p* = 0.024; Fig. 5i). Together, these data strongly suggest that the dopamine neurons have functioning glutamatergic synapses on the dendritic shaft.

### Different AMPAR/NMDAR current ratios between spine synapses and shaft synapses in the nigral dopamine neurons

It is very likely that the nigral dopamine neurons have both spine synapses and shaft synapses in substantial numbers on the same dendrite. Since AMPAR and NMDAR are two principal glutamate receptors and their current ratios are very important for synaptic functions, we next examined whether these two structurally different spine and shaft synapses are functionally similar, by examining their AMPAR/NMDAR current ratios. As shown in Fig. 6a, after visualizing dendrites of the nigral dopamine neurons with Alexa Fluor 594, spine and shaft synapses located close together on the same dendrite with similar distances from the soma (<100 μm) were examined. After establishing functional sites of the shaft and spine synapses (Fig. 6a, bottom, marked as two yellow dots), AMPAR- and NMDAR-mediated uEPSCs were measured by local glutamate uncaging at −60 mV and +40 mV, respectively (Fig. 6b). Although the NMDAR uEPSCs in spine synapses were higher than those of shaft synapses (at +40 mV, spine = 7.38 ± 0.99; shaft = 4.36 ± 1.00; *p* = 0.040; Fig. 6d), the AMPA uEPSCs in shaft synapses were higher than those of spine synapses (at −60 mV, spine = 5.88 ± 0.58; shaft = 10.01 ± 1.15; *p* = 0.003; Fig. 6d). Consequently, the ratio of AMPAR/NMDAR currents in the spine synapse was significantly lower than that in the shaft synapse (spine = 0.84 ± 0.05; shaft = 3.36 ± 0.47; *p* = 0.001; Fig. 6e). As shown in Fig. 6c, despite the wide distribution of each current point measured in different regions of different neurons (n = 16) in the AMPAR and NMDAR current distribution graph, there was a clear separation between the two different groups of synapses. The AMPAR/NMDAR current ratios within each group appear to be similar; in general the shaft synapses showed higher AMPAR/NMDAR current ratios, whereas the spine synapses showed lower AMPAR/NMDAR current ratios (Fig. 6e). These data clearly indicate that the spine synapses are functionally different from the shaft synapses.

Finally, we investigated whether the spine synapses and shaft synapses have a similar electrical impact on the soma, by measuring EPSPs evoked by local glutamate uncaging. As shown in Fig. 7, local glutamate uncaging was performed on spine and shaft synapses that were located at similar distances from the soma (red box in Fig. 7a). Although we were able to observe uEPSPs with a single pulse glutamate uncaging (1 ms duration), in most cases, 5 repetitive pulses (five pulses of 1 ms duration with 10 ms interval) were also applied for a clearer comparison. The amplitudes of uEPSPs from the spine synapses were lower than those of the shaft synapses (1P, spine = 0.84 ± 0.14; shaft = 1.48 ± 0.19; *p* = 0.024; Fig. 7b,c), but the decay time constants and half widths of the uEPSP in the spine synapses were significantly greater than those in the shaft synapses (1P, half width, spine = 31.84 ± 3.14; shaft = 17.80 ± 2.41; *p* = 0.008; 1P, τ, spine = 0.05 ± 0.003; shaft = 0.03 ± 0.002; *p* = 0.001; Fig. 7d,e). With 5 consecutive uncaging pulses, the slower decay of uEPSPS in the spine synapses was more pronounced (5P, half width, spine = 69.86 ± 7.00; shaft = 38.40 ± 4.39; *p* = 0.001; 5P, τ, spine = 0.55 ± 0.10; shaft = 0.21 ± 0.007; *p* = 0.012; Fig. 7d,e), but the amplitudes of uEPSPs were not dramatically exaggerated in contrast to the single pulse stimulation (5P, spine = 6.81 ± 0.55; shaft = 8.15 ± 1.10; *p* = 0.339; Fig. 7b), possibly because of local receptor saturation.

Thus, we conclude that the spine and shaft synapses in the nigral dopamine neurons might play functionally different roles as glutamatergic synapses. The nigral dopamine neuron appears to be a very particular type of neuron that has a substantial number of two distinct glutamatergic synapses - a spine synapse and a shaft synapse - together on the same dendrite.

## Discussion

It has been reported that dopamine neurons in the midbrain are involved in many kinds of brain function such as action selection, volitional movement, goal-oriented behavior, reinforcement learning, and reward processing^1^,^2^,^49^,^50^, as well as many neuropsychiatric diseases such as Parkinson’s disease, schizophrenia, and drug addiction^51^,^52^,^53^. Despite the wide involvement of dopamine neurons in various physiological functions and neuropsychiatric diseases, it has recently become clear that the roles of the dopamine neurons are, in general related to motivational states or reward information processing^49^,^50^. In animal experiments, it is well known that exposure to unexpected rewards or cues informing upcoming rewards causes dopamine neurons to generate burst firing through glutamatergic afferents^1^,^54^. In addition, the dopamine neurons receive glutamatergic afferent inputs from many parts of the brain and most of the excitatory inputs are glutamatergic afferents^49^,^50^,^55^. It is therefore very likely that significant portions of information determining reward value as well as salience are delivered to the dopamine neurons via glutamatergic synapses. Furthermore, glutamate signals in dopamine neurons are also involved in many kinds of drug addiction such as cocaine, heroin, and alcohol^55^. Therefore, glutamatergic synapses are of fundamental importance for understanding the physiological and pathological functions of the midbrain dopamine neurons. However, little is currently known about the morphology, distribution, and biochemical/electrical properties of glutamatergic synapses in the midbrain dopamine neurons. Since there has been no clear evidence for the presence of the functioning dendritic spines in midbrain dopamine neurons, they have been generally regarded as aspiny neurons and the importance of dendritic spines in the midbrain dopamine neurons has been largely neglected.

In our study of glutamatergic synapses on dopamine neuron dendrites, we were surprised to find that dopamine neurons have many spine-like structures, in contrast to the prevailing view that the midbrain dopamine neurons are aspiny^21^,^23^. High-resolution two-photon laser scanning confocal imaging showed several types of dendritic spines in the dopamine neurons including thin, mushroom, and stubby spines (Fig. 2). However, the number of dendritic spines of the dopamine neurons was approximately five times lower than that of CA1 pyramidal neurons measured in the same mice (Fig. 2). Interestingly, immunostaining and morphological analysis revealed more than twice as many glutamatergic synapses on the dendritic shaft without any membranous protrusion throughout the whole dendritic compartments (Fig. 3). Therefore, spine synapses and shaft synapses appear to be present together on the same dendrite with a similar distribution pattern (Fig. 2). Two principal glutamate receptors, AMPAR and NMDAR, were also found together in both the spine synapses and shaft synapses (Fig. 3). Using two-photon glutamate uncaging and patch-clamp techniques, we showed that these two morphologically distinct synapses differ in biophysical and electrical properties; the shaft synapses have higher AMPAR/NMDAR current ratios (Figs 4, 5, 6) and faster decay kinetics of EPSPs than the spine synapses (Fig. 7). Therefore, it appears that the dopamine neurons have a substantial number of two distinct glutamatergic synapses in the same dendritic region. It has generally been presumed that most of the central neurons exhibit one dominant type of glutamatergic synapse; either a spine synapse or a shaft synapse. In the hippocampal pyramidal neurons, cerebellar Purkinje neurons, and medium spiny neurons in the striatum, most glutamatergic signals occur in dendritic spines^14^,^15^, whereas most small interneurons and many GABAergic neurons appear to receive glutamatergic signals through aspiny shaft synapses^56^,^57^,^58^. In this respect, the dopamine neurons seem to be unique in receiving a substantial number of two distinct types of glutamatergic synapses on the same dendritic region. This finding could provide a very important basis to explain and understand many vital functions and pathological conditions of the dopamine neurons.

During our analysis of dendritic spines in the dopamine neurons, we found a substantial number of thin (36.4 ± 4.4%), mushroom (33.2 ± 5.0%), and stubby types of spines (26.7 ± 3.4%). In CA1 pyramidal neurons, thin, mushroom, and stubby type of spines accounted for 36.3 ± 4.6%, 33.9 ± 3.4% and 25.0 ± 3.2%, respectively (Fig. 2). Thus, although the number of dendritic spines in dopamine neurons was approximately five times lower than that of CA1 pyramidal neurons, the proportion of the three common shapes of spines was remarkably similar. However, the spines in dopamine neurons appear to be larger than those of CA1 pyramidal neurons (Fig. 2). This may, in part, be attributed to the larger soma size, thicker primary dendrites, and simpler dendritic arborization patterns of dopamine neurons (Fig. 1). Since the thin and mushroom spines are the two major types in dopamine neurons, our functional analysis was mostly performed on these two types of spine. As we did not find any significant functional differences between them (data not shown), in this study all of the electrical data obtained by two-photon glutamate uncaging were collectively presented as a representative spine in the dopamine neurons (Figs 4, 5, 6, 7).

During the analyses of synapse density, dendritic regions <80 μm from the soma were regarded as proximal dendrites, whereas the remote dendritic regions >80 μm were regarded as distal dendrites (Figs 1, 2, 3). The rationale for this classification is mostly based on our previous study^27^, in which the excitability and responsiveness of a dendrite to local glutamate uncaging differed significantly around the dendritic region 80 μm from the soma. Whereas the proximal dendritic regions in dopamine neurons are highly excitable and strongly participate in pacemaker activities, the distal dendrites are less excitable and do not participate in pacemaking activities^27^. We therefore compared spine densities between these functionally different parts of a dendrite.

Finally, it is worth mentioning the functional differences between the spine synapses and shaft synapses. There was a difference between these synapses in both the AMPAR/NMDAR ratio (Fig. 6) and the effect of EPSPs on the soma (Fig. 7). Because of the membranous protrusion of dendritic spines, the uEPSPs evoked by two-photon glutamate uncaging appear to be smaller than those of shaft synapses located at similar distances from the soma. However, the decay time constants of uEPSP in spine synapses were significantly longer than those of shaft synapses. Interestingly, when we stimulated with 5 consecutive pulses resembling a burst discharge, the decay time constant was increased dramatically in comparison with the increase in amplitude. This may be partly due to saturation of glutamate in the synaptic site and geometrical limits of spine synapses. Thus, these two spines may function differently if they receive burst types of glutamatergic afferent inputs. The physiological implications of the electrophysical differences between these two distinct synapses in dopamine neurons are not yet known and are focus of future research.

In summary, in this study, we provide the first clear evidence that dopamine neurons contain a substantial number of spine synapses through the whole dendritic compartment, together with more than twice as many shaft synapses. The specific organization of two distinct glutamatergic synapses on the same dendritic region could be a key basis for understanding the complex information processing of dopamine neurons.

## Materials and Methods

### Animals and preparation of midbrain slices

All experiments on animals were carried out in accordance with the approved animal care and use guidelines of the Laboratory Animal Research Center in Sungkyunkwan University School of Medicine and all experimental protocols were approved by the Laboratory Animal Research Center in Sungkyunkwan University School of Medicine. We purchased the STOCK Tg (TH-eGFP) DJ76Gsat/Mmnc line (NIH Mutant Mouse Regional Resource Centers), which was maintained as heterozygous mice by breeding with ICR(CrljOri: CD1) inbred mice. Horizontal midbrain slices containing the substantia nigra pars compacta (SNc) were prepared from postnatal day 21–28 transgenic mice expressing enhanced green fluorescent protein (eGFP) driven by the TH promoter. The transgene were identified by PCR on the genomic DNA extracted from toes. eGFP DNA was amplified by PCR, using the primers: for 5′-CCT ACG GCG TGC AGT GCT TCA GC-3′ and rev 5′ CGG CGA GCT GCA CGC TGC GTC CTC-3′. Actin DNA was amplified by PCR, using the primers: for 5′-GAT GAC GAT ATC GCT GCG CTG GTC G-3′, and rev 5′-GCC TGT GGT ACG ACC AGA GGC ATA CAG-3′. For live midbrain slices, mice were decapitated and the brains were removed rapidly. The midbrain section was sliced in ice-cold oxygenated artificial cerebrospinal fluid (ACSF in mM; 125 NaCl, 2.5 KCl, 10 Glucose, 1.25 NaH_2_PO_4_, mM NaHCO_3_, 1 MgCl_2_, 2 CaCl_2_, pH 7.4) using a vibratome (300 μm thickness, VT 1000S; Leica Microsystems, Germany). Midbrain slices were prepared in gassed (95% O_2_/5% CO_2_) ice-cold ACSF and allowed to recover in ACSF for 30 min at 33 °C before electrophysiological recordings. Slices were transferred to a small-volume (<1.5 ml) recording-chamber that was superfused continuously with ACSF saturated with 95% CO_2_ and 5% O_2_ at 33 °C and a flow rate of 2 ml/min. Unless otherwise mentioned, the bath solution also contained 1 μM tetrodotoxin (TTX), 100 μM CdCl_2_, 10 μM SR 95531, and 2 μM CGP 55845 to block spontaneous firing, voltage-operated calcium channels, GABA_A_- and GAB_AB_-receptors, respectively.

### Patch clamp recording

All experiments were performed at room temperature. Recording pipettes were made from thin-walled borosilicate glasses (TW150F-4, WPI, Sarasota, FL, USA; 3.5–4.5 MΩ) and filled with solution containing (in mM) 135 CsMeSO_3_, 4 NaCl, 10 Na_2_-phosphocreatine, 10 HEPES, 0.1 EGTA, 4 Mg-ATP, and 0.4 Na-GTP, with pH adjusted to 7.3 with CsOH. Osmolarity was adjusted to 295 mOsm. AMPAR-mediated EPSCs were recorded at −60 mV and NMDAR-mediated EPSCs were recorded at +40 mV in the voltage-clamp mode^46^. The peak of the AMPAR-mediated EPSCs was measured by the differences between the mean current amplitude over a 5 ms window around the peak and a 5 ms window on the baseline. The amplitude of the NMDAR-mediated uEPSCs was measured at 14 ms after onset, when AMPAR-mediated uEPSCs had sufficiently returned to baseline. Each current trace was averaged over 3 to 10 trials. Whole-cell voltage-clamp recordings were made with a HEKA EPC 10 amplifier. Electrical signals were filtered at 2 kHz and digitized at 20 kHz. Data analysis was performed with HEKA EPC programs and IgorPro software (Wavemetrics, Lake Oswego, OR, USA).

### Two-photon confocal imaging

Two-photon confocal imaging was performed using a Zeiss LSM510 scanning confocal/two-photon microscope system in the Research Core Facility of the Samsung Biomedical Research Institute (SBRI). Recorded neurons filled with Alexa Fluor 594 or Cascade blue were visualized maximally from the slice surface using a two-photon microscope with a pulsed Ti::sapphire laser (Mai Tai, Spectra Physics) tuned to 690–1020 nm (800 nm for fluorescence imaging of GFP and 720 nm for glutamate uncaging). Image stacks (512 × 512 pixels; 0.02 μm per pixel) were obtained with 0.3 to 1 μm z-steps. Most images shown are maximum projections of 3D image stacks after applying a low filter to the raw image data.

### Glutamate uncaging

MNI-caged-L-glutamate was dissolved in ACSF in the dark room at a concentration of 2.5 mM in 6 ml volumes. Stimulation was performed in a closed circulation system with continued carboxygenation. Excitatory postsynaptic currents (EPSCs) were evoked by local glutamate uncaging using a 1 ms pulse of the 720 nm beam, which was manually positioned 0.5 μm from the tip of the spine head or dendritic shafts. The amplitudes of AMPA-mediated uEPSCs at −60 mV were 1–20 pA, which were within the range of the miniature excitatory postsynaptic currents previously reported^44^. The laser power was adjusted to evoke a current of <20 pA. uEPSC amplitudes from individual spines and shafts were quantified and expressed as averages (3–10 test pulses). The uncaging spot size was 0.5 μm in diameter.

### Immunocytochemistry

Postnatal 21–28 day transgenic mice (TH-eGFP) were decapitated and the whole brain was quickly removed and chilled in ice-cold oxygenated HEPES-buffered saline (in mM: 135 NaCl; 5 KCl; 1 CaCl_2_; 1 MgCl_2_; 25 D-glucose; 10 HEPES; pH adjusted to 7.4 with NaOH). After sectioning of 300 μm coronal midbrain slices (VT 1000S, Leica Microsystems) containing the SNc, the region of the SNc demarcated by a dark color was dissected out using a scalpel blade and digested in O_2_ saturated HEPES-buffered saline containing papain (10 U/ml, Worthington Biochemical, Lakewood, NJ, USA) for 40 min at 37 °C. The tissue segments were subsequently rinsed with enzyme-free HEPES-buffered saline and then gently triturated with a fire-polished micro-Pasteur pipette. Acutely isolated single dopamine neurons were fully attached for 1 hr on poly-D-lysine coated coverslips and preincubated in 10 N HCl for 1–4 hr at 80 °C. Cells attached to glass coverslips were fixed with 4% sucrose in 4% paraformaldehyde for 20 min at 4 °C. After fixation, the cells were washed with 1× phosphate-buffered saline (PBS) three times for 10 min, permeabilized with 1% BSA and 0.1% Triton X-100 in 1× PBS for 5 min, and then incubated with primary antibodies overnight at 4 °C. After extensive washing with 2% normal goat serum (NGS) in 1× PBS, appropriate secondary antibodies were applied at 1:200 or 1:100 dilution for 2 hr at room temperature.

Surface AMPA and NMDA receptors were also measured as described by Wei *et al*.^59^. Acutely dissociated cells were fixed with 4% paraformaldehyde in 4% sucrose contained 1x PBS (30 minute, RT) but not permeabilized. Cells were washed with 1x PBS three times for 10 min, incubated with 2% NGS in 1x PBS, and then incubated with extracellular binding 1^st^ anti-bodies of anti-GluR1 (Alomone labs, Jerusalem, Israel) or anti-GluN1 (synaptic systems, Goettinggen, Germany) were applied for 2 hr at room temperature. After extensive washing with 2% NGS in 1x PBS, cells were permeabilized with 0.1% Triton X-100 in 2% NGS for 5 min. After washing with 2% NGS, cells were incubated with anti-tyrosine hydroxlase antibody for overnight at 4 °C. Tyrosine hydroxlase was detected with Alexa Fluor 488 (green)-conjugated anti-rabbit 2^nd^ antibody, whereas surface GluR1 or GluN1 were detected with Alexa-635 (red)-conjugated anti-mouse 2^nd^ antibody. Fluorescence of secondary antibodies was obtained using a Zeiss 510 confocal laser scanning microscope (Carl Zeiss AG, Germany).

### Quantitative analysis of spine density and 3D reconstruction of dendrite morphology

All images were digitized under the same illumination conditions at a resolution of 512 × 512 pixels. The two-photon z-stacks were imported to Imaris x64 for three-dimensional (3D) reconstruction of spine and dendrite. The spine density was calculated by counting spine numbers of each dendritic segment. A spine was labeled as a thin type if it had head <0.7 μm in diameter and a maximum length that was at least twice the head diameter. A spine was classified as a mushroom spine if the head diameter exceeded 0.7 μm. Stubby spines were classified based on the absence of a neck^60^. Other spine-like structures different from the spines described above were categorized as unidentified or unclassified spines.

### Statistics

Statistical analysis was performed with unpaired Student’s t-test and one-way ANOVA. *P*-values < 0.05 were regarded as significant. Data are represented as mean ± standard error of the mean (SEM).

## Additional Information

**How to cite this article**: Jang, M. *et al*. Coexistence of glutamatergic spine synapses and shaft synapses in substantia nigra dopamine neurons. *Sci. Rep.* **5**, 14773; doi: 10.1038/srep14773 (2015).

## Supplementary Material

Supplementary Information
