# Supplementary material for: Coexistence of glutamatergic spine synapses and shaft synapses in substantia nigra dopamine neurons
Source: Sci Rep. 2015 Oct 5;5:14773. doi: 10.1038/srep14773 (PMC4593176; doi:10.1038/srep14773)
Supplement: Supplementary Information [file srep14773-s1.pdf]

Supplementary Information for

Coexistence of glutamatergic spine synapses and shaft synapses in substantia nigra dopamine neurons

Miae Jang[1,2ψ], Ki Bum Um[1ψ], Jinyoung Jang[1,2], Hyun Jin Kim[1,2], Hana Cho[1,2], Sungkwon Chung[1,2], Myoung Kyu Park[1,2*]

[1]Department of Physiology, Sungkyunkwan University School of Medicine 300 Chunchun-dong, Jangan-ku, Suwon, 440-746, Korea
[2]Center For Molecular Medicine, Samsung Biomedical Research Institute, 300 Chunchun-dong, Jangan-ku, Suwon, 440-746, Korea

*To whom correspondence should be addressed.
TEL: +82-31-299-6101; FAX: +82-31-299-6129; e-mail: mkpark@skku.edu

Table of contents

Supplementary Figures S1-3 and Figure Legends

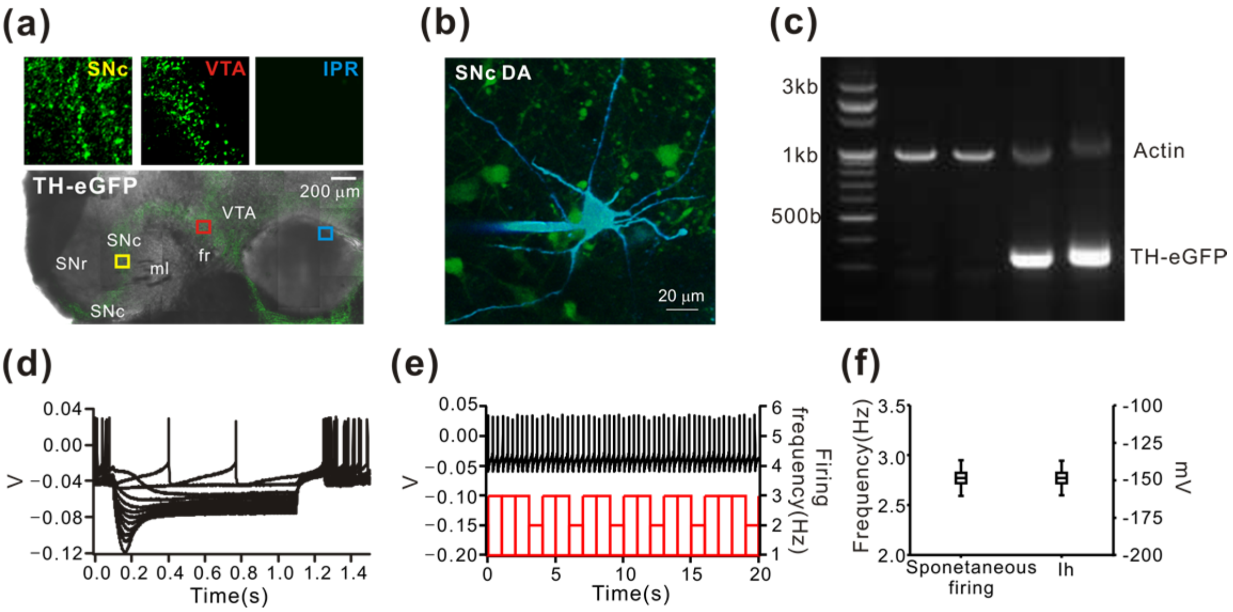

Figure S1. Molecular and electrophysiological properties of GFP-expressing cells in TH-eGFP Mice. **(a)** Fluorescence image of GFP-expressing cells in midbrain slices of TH-eGFP mice. SNc, substantia nigra compacta; VTA, ventral tegmental area; ml, medial lemniscus; SNr, substantia nigra reticular; fr, fasciculus retroflexus; IPR, interpedunc nu, rostral sub. **(b)** Fluorescence image of a nigral dopamine neuron labeled with cascade blue. **(c)** PCR of DNA for identification of TH-eGFP mice. **(d-e)** Whole-cell patch-clamp recordings from GFP-expressing dopamine neurons. Membrane potentials in response to hyperpolarizing currents and spontaneous firing, respectively. **(f)** Graphs showing mean peak currents of sag in response to hyperpolarizing currents, -300 pA and mean spontaneous firing frequency (n=12). In TH-eGFP neurons in the SNc the spontaneous firing rate (2.76 ± 0.17), peak of Ih (-148.65 ± 11.53), and specific delayed responses at the end of hyperpolarizing step pulses were identical with those of wild-type dopamine neurons (firing frequency = 2.61 ± 0.36; peak Ih = -144.43 ± 7.69; n=6).

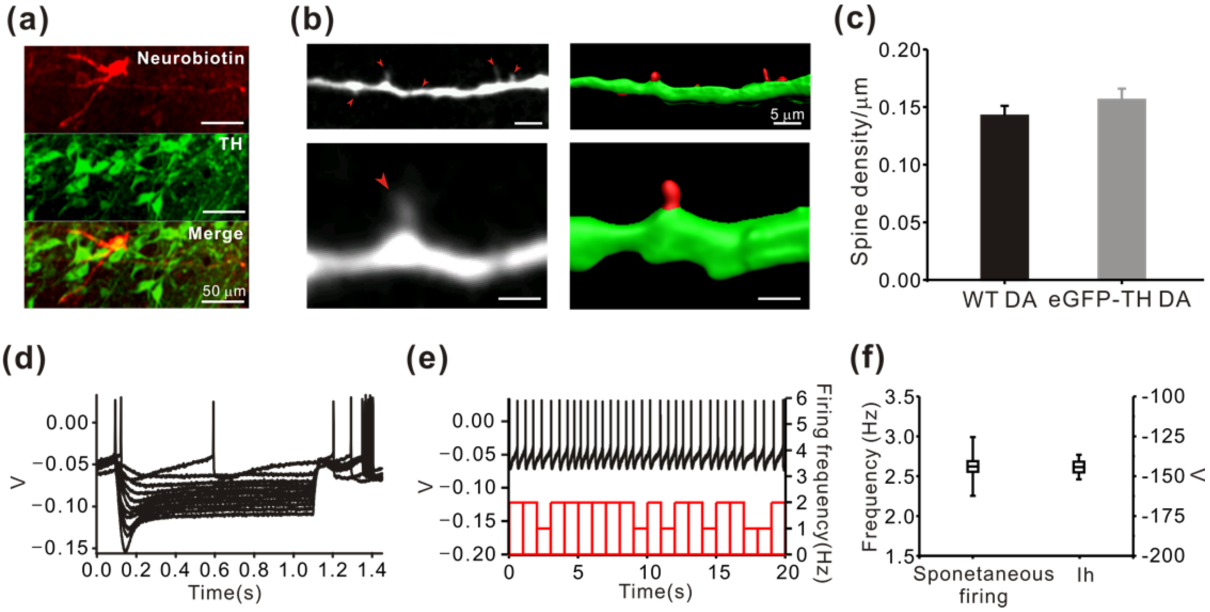

Figure S2. Spine density and electrophysiological properties of dopamine neurons in wild-type mice are identical with GFP-expressing cells in TH-eGFP mice. **(a)** Immunostaining with antibodies specific for TH of wild-type dopamine neurons that were filled with neurobiotin via the recording pipette. **(b)** Monochrome and three-dimensional (3D) reconstructed images of the dendritic segment (green and red images; shafts are green and spines are red). Red arrows indicate spines. **(c)** Comparison of mean spine densities between wild-type dopamine neurons (WT SNc = 0.14 ± 0.008, 10 dendrites in 4 cells) and GFP-expressing neurons (TH-eGFP SNc = 0.15 ± 0.009). **(d-e)** Whole-cell patch-clamp recordings from GFP-expressing dopamine neurons. Membrane potentials in response to hyperpolarizing currents and spontaneous firing, respectively. **(f)** Graphs showing mean spontaneous firing frequency and mean peak current of sag in response to hyperpolarizing current, -300 pA (n=6).

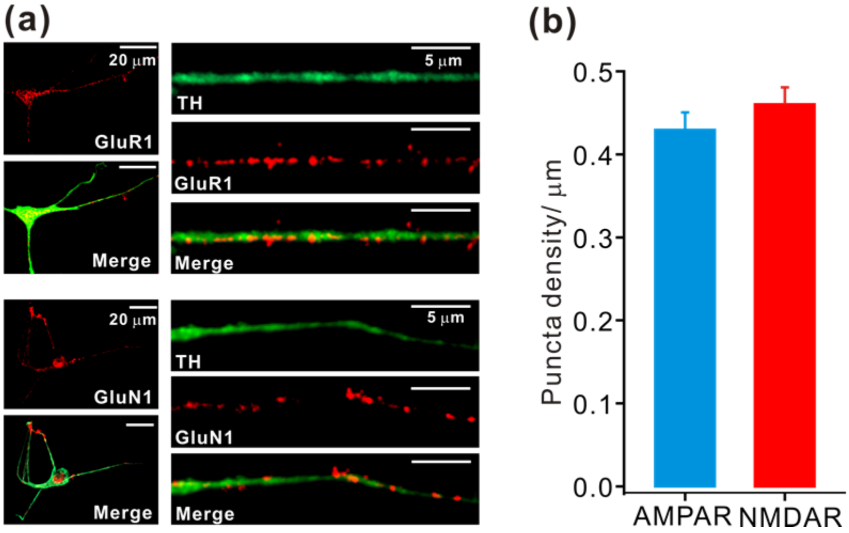

Figure S3. Molecular identification of surface AMPA and NMDA receptors in the dendrites of SNc dopamine neurons. **(a)** Distribution of AMPA receptors and NMDA receptors in the dendritic shafts of SNc dopamine neurons. Double immunostaining of dissociated dopamine neurons with TH and either surface-expressed GluR1 for AMPAR or GluN1 for NMDAR. TH (green), GluR1 or GluN1 (red), and their overlapped images (merge) are presented. **(b)** GluR1 puncta (from 26 dendrites of 23 cells; 0.46 ± 0.02) and GluN1 puncta (from 30 dendrites of 21 cells; 0.46 ± 0.02) measured only in the dendritic shaft from 3 mice. Error bars indicate SEM.
